# Case Report: Atypical neuroleptic malignant syndrome induced by paliperidone palmitate—diagnostic challenges and clinical considerations

**DOI:** 10.3389/fpsyt.2026.1718815

**Published:** 2026-02-11

**Authors:** Omar El Oumary, Hicham Laaraj, Mina Ouhamou, Khalid Mouhadi, Jalal Doufik, Khadija Akebour, Saliha Hamri, Ismail Rammouz

**Affiliations:** 1 Department of Psychiatry, Faculty of Medicine, Ibn Zohr University, Agadir, Morocco; 2 Innovation and Ethics in Clinical Neuroscience (NICE), Research Laboratory “Kidney, Endocrinology, Gastroenterology, Neurosciences and Ethics” (REGNE), Faculty of Medicine, Ibn Zohr University, Agadir, Morocco

**Keywords:** atypical presentation, delayed-action preparations, neuroleptic malignant syndrome, paliperidone palmitate, schizophrenia

## Abstract

Poor treatment adherence is common in schizophrenia and constitutes a major risk factor for relapse and clinical instability. Long-acting injectable (LAI) antipsychotics are frequently prescribed to mitigate this risk; however, their use can be associated with rare but serious adverse events, including neuroleptic malignant syndrome (NMS), with certain atypical presentations posing considerable diagnostic challenges. We report the case of a 51-year-old patient with schizophrenia who, during hospitalization for a psychotic relapse following prolonged refusal of all oral medications, received an intramuscular injection of paliperidone palmitate. Within a few days, the patient experienced a rapid clinical deterioration, characterized by hyperthermia (38 °C), tachycardia (120 bpm), generalized muscular rigidity, mutism, confusion, dehydration, and refusal of oral intake. No obvious precipitating factors, including infectious or environmental triggers, were identified. Initial laboratory workup revealed marked creatine phosphokinase elevation (1700 U/L), hyperferritinemia, and evidence of a pulmonary infiltrate on chest imaging. Given this constellation of clinical and laboratory findings, a diagnosis of neuroleptic malignant syndrome (NMS) was considered. The lack of improvement with antibiotic therapy alone, the persistence of extrapyramidal symptoms, and the ongoing laboratory abnormalities supported the diagnosis of an atypical form of NMS. The patient was transferred to the intensive care unit, where symptomatic management—including intravenous rehydration, antipyretics, empirical antibiotic therapy, and diazepam—resulted in gradual clinical improvement. Complete recovery was achieved after five weeks of hospitalization. Antipsychotic therapy was cautiously reintroduced with olanzapine, which was well tolerated and not associated with any recurrence of symptoms. This case illustrates the diagnostic and therapeutic challenges of neuroleptic malignant syndrome associated with long-acting injectable antipsychotics and emphasizes the need for close and prolonged monitoring in patients receiving these formulations.

## Introduction

Schizophrenia is a severe and chronic psychiatric disorder affecting more than 23.18 million people worldwide (1). It is associated with a marked reduction in life expectancy and profound functional impairment, particularly in social and family domains (2). The course of the illness is generally characterized by periods of remission interspersed with symptomatic exacerbations, which may require repeated hospitalizations (3).

Treatment primarily relies on antipsychotic medications, which constitute the cornerstone of schizophrenia management by reducing symptom severity, improving overall functioning, and preventing relapse (4). However, their effectiveness is frequently compromised by poor treatment adherence, which remains a major determinant of relapse and recurrent hospitalizations (3).

To address this challenge, second-generation (atypical) antipsychotics are recommended as first-line treatment for schizophrenia because of their more favorable tolerability profile (5); however, this does not consistently ensure sustained adherence. In this context, long-acting injectable (LAI) formulations represent a promising strategy by improving adherence, significantly reducing relapse rates, and decreasing hospitalization rates (6).

Among these agents, paliperidone palmitate—an active metabolite of risperidone—represents an effective and well-tolerated option for both the treatment of the acute phase and maintenance therapy (7). Consequently, long-acting atypical antipsychotics are recommended by current clinical guidelines for patients with poor treatment adherence (8).

Despite their substantial therapeutic benefits, both typical and atypical antipsychotics may induce severe adverse effects, among which neuroleptic malignant syndrome (NMS) remains one of the most feared complications (9). Although rare, NMS constitutes a potentially life-threatening medical emergency, with an estimated incidence ranging from 0.01% to 0.02% among patients receiving these agents (10). Available data suggest that second-generation (atypical) antipsychotics may be associated with a comparatively lower risk of NMS (11).

However, the increasing number of reported cases related to these agents underscores the need for heightened clinical vigilance regarding their manifestations (12). Accordingly, early recognition of the syndrome, including in patients treated with newer-generation atypical antipsychotics such as paliperidone, remains a clinical priority.

In this context, we report a rare clinical case of neuroleptic malignant syndrome occurring after the administration of paliperidone palmitate in a patient with schizophrenia, illustrating the diagnostic and therapeutic challenges posed by atypical presentations induced by long-acting injectable antipsychotics.

## Clinical case

We report the case of a 51-year-old married male patient, father of two children, diagnosed with schizophrenia with symptom onset at age 15. The patient had no history of somatic medical conditions. However, his family history was notable for schizophrenia in his mother and two siblings. He had no history of substance use or suicide attempts.

Regarding his educational background, the patient left school shortly after the onset of psychotic symptoms and was never able to maintain stable employment. No neurodevelopmental disorders were reported, and he had no history of medication intolerance or extrapyramidal symptoms during prior treatments.

Although the initial psychotic symptoms developed insidiously, therapeutic management was initiated only three years later, after a clearly defined first psychotic episode characterized by polymorphic, non-systematized delusions, auditory hallucinations, and behavioral disorganization. This episode necessitated hospitalization and pharmacological treatment. The initial response to antipsychotic therapy was obtained with haloperidol (6 mg/day) and levomepromazine (100 mg/day), resulting in partial symptom remission.

Nevertheless, the clinical course was characterized by multiple recurrent hospitalizations, each consistently preceded by a gradual discontinuation of pharmacological treatment, highlighting persistent poor treatment adherence. This non-adherence represented a key factor in symptomatic relapses, in the absence of any identifiable iatrogenic exacerbation or environmental trigger.

At the age of 34, the clinical presentation worsened with the onset of a major depressive episode characterized by persistent depressed mood and suicidal ideation. Treatment with clomipramine (150 mg/day) led to partial improvement in mood, without complete resolution of residual psychotic symptoms.

Nine months prior to the most recent admission, the patient again discontinued all medications, leading to a severe psychotic relapse over a three-month period, characterized predominantly by persecutory delusions, auditory hallucinations, hetero-aggressive agitation, sleep disturbances, and persistent treatment refusal.

In response to this relapse, psychiatric hospitalization was required. Upon admission, a treatment regimen was initiated, combining risperidone (4 mg/day), lorazepam (7.5 mg/day), and zopiclone (7.5 mg/day). The patient initially accepted and adhered to this treatment for one week.

However, the patient rapidly exhibited a persistent refusal to take any oral medication. This refusal lasted for four days, during which no pharmacological treatment could be administered, thereby compromising the desired clinical stability. In response to this evident non-adherence and to ensure minimal therapeutic continuity, treatment with a long-acting injectable formulation—specifically paliperidone palmitate monotherapy—was initiated as a monthly injection.

The clinical course was marked by persistent psychiatric symptoms and a deterioration of the patient’s general condition, occurring three days after administration of paliperidone palmitate. Clinical features included fever of 38 °C, sweating, dehydration, tachycardia at 120 bpm, mild mental confusion, rigidity and hypertonia of the limbs, mutism, negativism, and refusal to eat.

The patient’s history revealed no contact with individuals with infectious diseases, either at home or within the psychiatric unit. Moreover, no other signs of infection—such as anosmia, dyspnea, or gastrointestinal symptoms—were observed. Oxygen saturation was normal, and no major environmental factors likely to precipitate clinical destabilization, such as dehydration or prolonged agitation, were identified.

Given this suggestive symptomatology, neuroleptic malignant syndrome (NMS) was suspected, and the patient was transferred to the intensive care unit for specialized management. Biological and radiological investigations were performed. Results revealed a markedly elevated creatine phosphokinase (CPK) level of 1700 IU/L. Transaminases, bilirubin, and lactate dehydrogenase (LDH) were at the upper limit of normal. Complete blood count showed normochromic normocytic anemia (hemoglobin 10 g/dL), thrombocytopenia (128,000/mm³), and a normal leukocyte count.

Serum electrolytes and renal function were within normal limits. However, serum ferritin was markedly elevated at 1702 µg/L. An infectious workup showed increased C-reactive protein (CRP) and procalcitonin levels, along with chest radiography and computed tomography findings consistent with pneumonia. Acid-fast bacilli testing on three bronchial specimens was negative. TPHA/VDRL and HIV serologies were also negative. D-dimer levels were within the normal range.

The patient received intravenous rehydration, antipyretic treatment with intravenous paracetamol, broad-spectrum antibiotic therapy with amoxicillin-clavulanic acid for pneumonia, and anxiolytic treatment with intramuscular diazepam. A follow-up chest CT scan performed seven days after hospitalization demonstrated complete resolution of pulmonary lesions. However, the patient’s rigidity and fever only began to subside on day 10, accompanied by a gradual decline in creatine phosphokinase (CPK) levels, which normalized fully after 35 days, thereby supporting the diagnosis of neuroleptic malignant syndrome.

Two weeks after resolution of the neuroleptic malignant syndrome episode, antipsychotic therapy was cautiously reintroduced, starting with olanzapine at 5 mg/day and gradually titrated to 10 mg/day. This reintroduction was associated with marked clinical improvement. At six-month follow-up, the patient remained psychiatrically and somatically stable, with good adherence to olanzapine therapy (10 mg/day).

**Figure 1:**
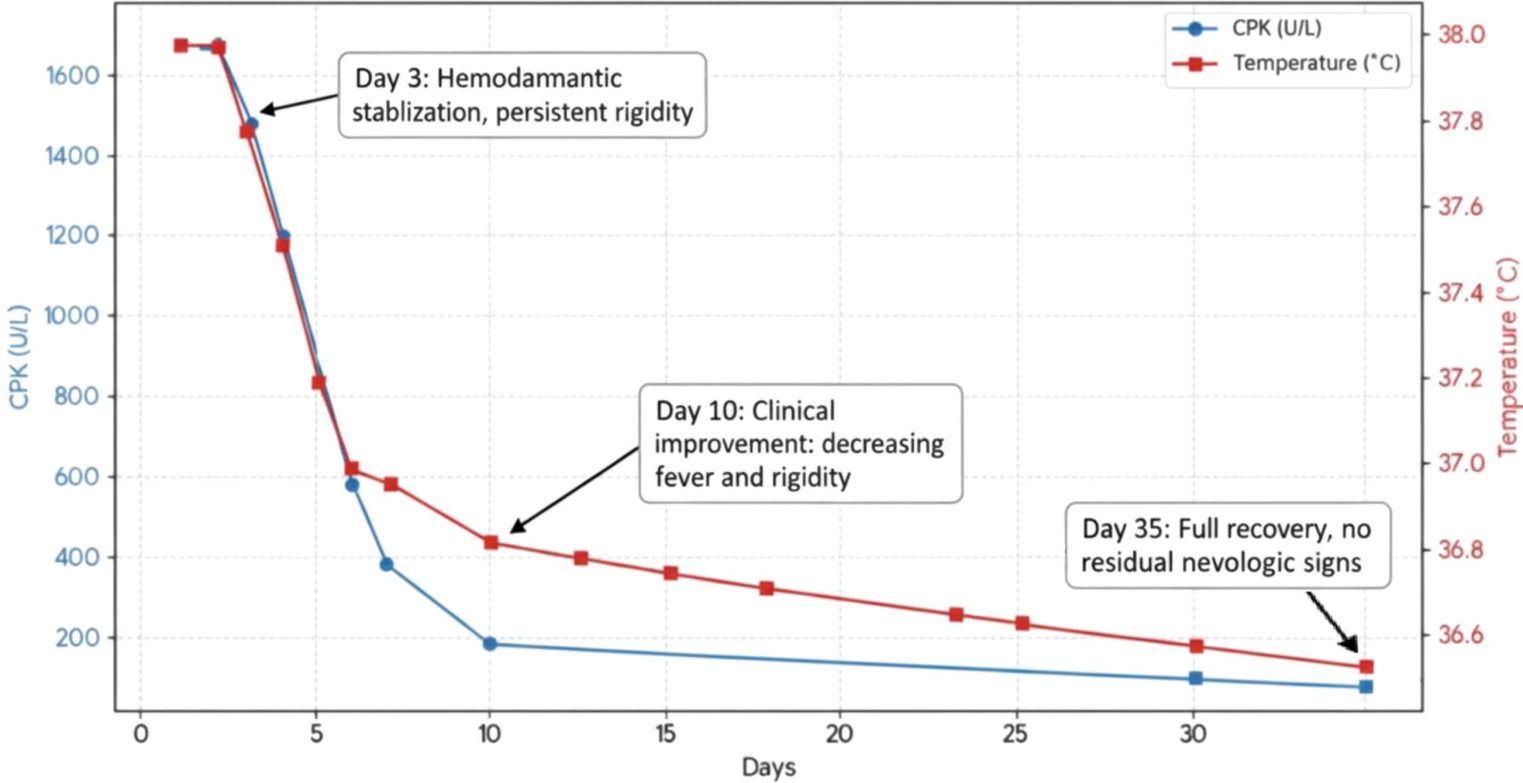
Evolution of clinical and biological parameters in a patient with neuroleptic malininant syndrome.

The history of pharmacological treatments and clinical responses are summarized in **Table 1**, whereas **Figure 1** provides an overview of Evolution of clinical and biological parameters in a patient with neuroleptic malininiant syndrome.

**Table 1:** History of pharmacological treatments and clinical response.

Treatment duration	Pharmacological treatment	Symptoms and treatment response
Since adolescence to 2021 (recurrent episodes)	Haloperidol 6 mg/day, Levomepromazine 100 mg/day	Initial partial response with psychotic symptom relief. Longstanding poor adherence, multiple relapses.
2002–2010	Clomipramine 150 mg/day (add-on)	Partial improvement in depressive symptoms, residual psychotic features persisted.
2021—1 week before treatment refusal	Risperidone 4 mg/day, Lorazepam 7.5 mg/day, Zopiclone 7.5 mg/day	Mild initial improvement. Severe psychotic relapse (persecutory delusions, auditory hallucinations, agitation). Early oral treatment refusal.
April 2021—1 injection	Paliperidone palmitate (PP1M) 150 mg i.m.	Fever (38 °C), rigidity, confusion, mutism, tachycardia, dehydration. Concurrent pneumonia. Atypical Neuroleptic Malignant Syndrome. ICU admission. Gradual resolution over 35 days.
15 days after NMS resolution	Olanzapine 5 mg/day up to 10 mg/day	Well tolerated. Marked clinical improvement. Full psychiatric and physical stability at 6-month follow-up.

## Discussion

Neuroleptic malignant syndrome (NMS), a rare but severe medical emergency historically associated with typical antipsychotics, has been increasingly reported with atypical antipsychotics, which may present with non-classical or partial manifestations along the neuroleptic malignant syndrome spectrum, characterized by incomplete or attenuated symptom expression, thereby complicating the diagnostic process (10).

In response to this clinical variability, Trollor et al. proposed an expanded definition of atypical neuroleptic malignant syndrome, encompassing clinical presentations that do not simultaneously fulfill all four major criteria: hyperthermia, generalized muscle rigidity, altered mental status, and autonomic instability (13).

The need for early detection remains particularly critical in light of the growing use of long-acting injectable (LAI) formulations, such as paliperidone palmitate (PP1M), whose adverse effects, although rare, may be severe—notably neuroleptic malignant syndrome (NMS). These manifestations remain poorly characterized in the literature, where they have been reported only exceptionally, with an estimated incidence of approximately 4 per 10,000 patient-years (14).

The analysis of published cases of neuroleptic malignant syndrome (NMS) over the past two decades highlights distinct trends in terms of clinical profiles, severity, and management according to the long-acting injectable (LAI) antipsychotic involved. Cases associated with first-generation LAIs (fluphenazine, flupentixol, zuclopenthixol, haloperidol) and with risperidone LAI (**Table 2**) exhibit a broader and more variable clinical spectrum, including atypical or afebrile presentations and wide variability in creatine phosphokinase (CPK) elevations. In contrast, cases involving paliperidone palmitate (**Table 3**) are generally more severe, often presenting with markedly higher CPK elevations, with several cases exceeding 10,000 IU/L, reflecting a potentially more intense muscular response and rhabdomyolysis. A notable common feature between the two groups is the timing of NMS onset, frequently observed around the 7th day after injection, highlighting a critical period of vulnerability related to LAI pharmacokinetics. From a therapeutic perspective, cases related to both first-generation LAIs and paliperidone palmitate benefited from similar interventions (bromocriptine, benzodiazepines, dantrolene). However, admissions to intensive care units (ICUs) and the use of complex treatments (dialysis, correction of acidosis) were more frequent in cases associated with paliperidone palmitate due to greater biological severity.

**Table 2:** Summary of published cases of neuroleptic malignant syndrome associated with long-acting injectable antipsychotics.

Reference (authors, year)	Age/Sex	Main clinical features	Formulation	Time to nms onset (days)	Cpk (U/L)	Treatment	Outcome/Course
Dragonetti et al., 2024 (29)	23/M	Fever, tachycardia, tachypnea, rigidity	Fluphenazine decanoate (LAI)	7	Elevated creatine kinase	Benzodiazepines, dantrolene, ECT	Improvement, with persistent bradykinesia and tremors
Elyasi et al., 2023 (28)	46/M	Generalized rigidity, negativism, neck stiffness	Flupentixol (LAI)	7	1,476	Bromocriptine and lorazepam	Improvement
Mędrala et al., 2023 (30)	32/F	Altered consciousness, muscle rigidity, fever, vegetative disturbances	Zuclopenthixol decanoate (LAI)	7	2,615	Benzodiazepines and ECT	Improvement
Aki et al., 2022 (31)	23/M	Dysarthria, dysphagia, hypersudation	Zuclopenthixol (LAI)	14	485	Benzodiazepines, bromocriptine, dantrolene, ECT	Complete recovery, no residual sequelae
Chen et al., 2018 (32)	47/F	Altered consciousness, muscle rigidity, dysphagia	Risperidone (LAI)	—	3,196	Lorazepam	Improvement, then switched to Paliperidone LAI
Mitchell et al., 2017 (33)	66/M	Hypotension, fever, diarrhea, vomiting	Risperidone (LAI)	7	783	—	Improvement after a few days
Khouri et al., 2016 (34)	55/F	Hyperthermia, limb tremors, mild rigidity	Risperidone (LAI)	14	256	Dantrolene	Symptom improvement after 15 days
Yamashita et al., 2013 (35)	50/M	Fever, muscle rigidity, tremors, dysphagia, tachycardia, altered consciousness	Risperidone (LAI) + oral risperidone + multiple psychotropics	7	1089	Discontinuation of antipsychotics, supportive care, ICU management	Gradual improvement
Assareh & Habibi, 2010 (36)	41/M	Altered consciousness, muscle rigidity, waxy flexibility, mutism, severe diaphoresis, tachycardia	Fluphenazine decanoate (LAI)	14	2800	Lorazepam	Improvement after 15 days of hospitalization
Varoglu et al., 2009 (37)	60/M	Disturbed consciousness, fever, hypertonicity, hypotension. Hyperglycemia, suspected acute myocardial infarction.	Zuclopenthixol IM (LAI)	4	428	Discontinuation of antipsychotics, IV hydration, bromocriptine, diazepam	Sudden death due to cardiogenic shock 3 days after admission
Erermis et al., 2007 (38)	14/F	Change in mental status, diffuse muscular rigidity, catatonic excitement/posturing, hypertonia, tremor. NMS without hyperthermia	Zuclopenthixol-decanoate IM (LAI)	2	2300	Bromocriptine and diazepam	Improvement within 7 days. Full recovery
Tiryaki et al., 2006 (39)	42/M	Unconsciousness, autonomic irregularity, fever, leukocytosis. Symptoms consistent with NMS and Serotonin Syndrome	Flupenthixol depot (LAI) + Zuclopenthixol depot (LAI) + Citalopram (Oral)	1	2813	Supportive care, Bromocriptine	Regained consciousness on Day 8. Discharged on Day 12. Full recovery
Tadke & Suryavanshi, 2006 (40)	22/F	Altered behavior, fever, rigidity, tremors, incontinence, autonomic disturbances	Haloperidol IM	A few days	3850	Intravenous Valproate; Clonazepam	Dramatic improvement observed by the next day. Full recovery

**Table 3:** Summary of published cases of neuroleptic malignant syndrome (NMS) associated with paliperidone palmitate.

Reference (authors, year)	Age/Sex	Main clinical features	Formulation	Time to NMS onset (days)	CPK (U/L)	Other notable features	Treatment	Outcome/Course
Unal et al., 2025 (12)	27/M	Fever, generalized rigidity, altered consciousness, dysautonomia	Paliperidone palmitate PP1M	5	18000	No concomitant antipsychotic use; initial differential diagnosis with encephalitis	IV dantrolene, bromocriptine, intensive care	Full recovery after 12 days
Edinoff et al., 2022 (41)	34/M	Confusion, altered mental status, psychotic regression, disorientation, thought blocking	Risperidone PO, Aripiprazole PO, Paliperidone PO then Paliperidone palmitate PP1M, Haloperidol IM	Several days	7101	Schizophrenia + mild intellectual disability, haloperidol IM after deterioration, lithium added	Discontinuation of antipsychotics, quetiapine, lithium, IV hydration	Rapid improvement, clinical stabilization, discharged on quetiapine and lithium
Yeung et al., 2021 (42)	72/F	Acute altered mental status, mutism, generalized rigidity, hyperreflexia, hyperthermia, fluctuating BP	Haloperidol PO, Paliperidone Palmitate PP1M	14	276	Advanced Lewy body dementia, UTI, suspected aspiration pneumonia	Dantrolene, bromocriptine, benzodiazepines, amantadine, antibiotics	Rigidity improved, autonomic signs stabilized, neurological status unchanged. ICU.
Netcheva & Shin, 2021 (43)	63/F	Fever, rigidity, mutism, tachycardia	Paliperidone palmitate PP1M	3	9200	History of NMS under haloperidol; recent reintroduction of LAI	Dantrolene, bromocriptine, benzodiazepines	Complete clinical recovery
Kane et al., 2019 (14)	40/M	Fever, rigidity, confusion, tremor	Paliperidone palmitate PP3M	10	14000	Recent switch from PP1M to PP3M; dose increase	Dantrolene, bromocriptine, rehydration	Complete recovery
Langley-DeGroot et al., 2016 (10)	31/M	Fever, rigidity, severe dysautonomia, mutism	Paliperidone palmitate PP1M	7	11800	Rapid dose escalation; no concomitant neuroleptics	Dantrolene, amantadine, intensive care	Recovery without sequelae
Kaur et al., 2016 (44)	35/M	High fever, severe muscle rigidity, upper limb contractures, tonic-clonic seizures, altered consciousness, severe hyponatremia, metabolic acidosis, tachycardia.	Paliperidone palmitate PP1M	3	34548	Paliperidone-induced SIADH, hypo-osmolality, rhabdomyolysis, liver enzyme elevation, elevated lactate, EEG diffuse dysfunction, intubation	Hypertonic saline, IV hydration, sodium bicarbonate, dantrolene IV, bromocriptine, lorazepam, sodium correction	Gradual improvement, lab normalization in 2 weeks, transferred to psychiatric unit stable
Nayak et al., 2011 (45)	34/F	Fever, rigidity, excessive sweating, altered alertness	Paliperidone palmitate PP1M	3	10500	Combined with lithium	Dantrolene, bromocriptine, lithium discontinuation	Gradual improvement
Patel & Brunetti, 2010 (46)	37/F	Fever, rigidity, tachycardia, sweating, confusion	Paliperidone palmitate PP1M	5	12000	Recent dose increase; obesity	Dantrolene, bromocriptine	Improvement under treatment

The present case exhibits several features that distinguish it from previously published reports (**Tables 2**, **3**). First, NMS onset occurred only three days after PP1M injection, representing a particularly short latency and placing it among the earliest documented cases. Second, in contrast to the majority of reported cases in which CPK levels exceeded 10,000 IU/L, this observation was characterized by a moderate elevation of CPK. Finally, the concomitant presence of confirmed pneumonia, elevated systemic inflammatory markers, and marked hyperferritinemia constitutes a distinct and complex biological profile, underscoring a major divergence from the existing literature.

In this case, the administration of PP1M—initiated due to poor treatment adherence—was followed, after a latency period, by a clinical presentation consistent with neuroleptic malignant syndrome (NMS), including confusion, mutism, muscular rigidity, hyperthermia, and autonomic instability. An elevation of creatine phosphokinase (CPK) to 1,700 IU/L further supported the diagnosis.

Although certain elements of the clinical presentation could initially suggest a classic form of NMS, a comprehensive analysis of the diagnostic criteria—taking into account the pharmacological context, the temporal course, and the semiological features—supports the interpretation of this presentation as part of a non-classical expression within the neuroleptic malignant syndrome spectrum. The patient exhibited moderate hyperthermia not exceeding 38 °C, marked dysautonomia, and a relatively moderate alteration of mental status (mutism, negativism, and mild confusion, without deep coma or severe agitation), along with elevated CPK levels, in the absence of high-grade fever and severe or generalized muscular rigidity. This presentation illustrates a partial and attenuated expression within the NMS spectrum, rather than a deviation from the syndrome itself, and contrasts with the classic NMS phenotype described in early clinical observations (temperature ≥ 39 °C, “lead-pipe” rigidity, and massive CPK elevation) (15).

This semiological dissociation corresponds to infra-threshold or subacute forms of NMS, as described by Trollor et al., which are more frequently associated with second-generation antipsychotics, occur often in older patients, and are characterized by an attenuated expression of motor and thermoregulatory manifestations (13, 16). Data from the systematic review by Singhai et al. further confirm that atypical NMS may present with suggestive biological and clinical abnormalities despite the absence of extreme rigidity or marked hyperthermia (16). The lack of validated diagnostic criteria for these non-classical presentations exposes clinicians to a risk of under- or overdiagnosis, underscoring the need for an integrative clinical approach and more sensitive diagnostic criteria to improve the recognition of atypical NMS in clinical practice.

In our case, chest radiography and thoracic computed tomography revealed pneumonia associated with a marked elevation of inflammatory biomarkers (C-reactive protein and procalcitonin), initially suggesting an infectious etiology for the febrile presentation. However, the temporal evolution of the clinical manifestations—characterized by rapid resolution of the pulmonary focus under antibiotic therapy—contrasted with the prolonged persistence of fever, muscular rigidity, autonomic instability, and elevated CPK levels. This temporal dissociation, confirmed by radiological normalization as early as day 7 while muscle enzyme levels stabilized only by day 35, suggests that the pulmonary infection was more likely an intercurrent event—possibly aspiration pneumonia or an opportunistic superinfection—rather than a primary infectious process accounting for the entire clinical picture. Accordingly, although intercurrent somatic comorbidities initially complicated the diagnostic assessment, the persistence of systemic and neuromuscular signs despite complete resolution of the infectious focus, together with the delayed and progressive normalization of CPK, constitutes a decisive argument in favor of atypical neuroleptic malignant syndrome rather than a classic form complicated by infection.

From a biological standpoint, although CPK levels were elevated (1,700 IU/L), they did not reach the thresholds typically observed in fulminant forms, suggesting moderate rhabdomyolysis. Moreover, hyperferritinemia (1,702 μg/L), while nonspecific, may reflect the severity of the inflammatory response and muscle breakdown and warrants further investigation as a potential biomarker of NMS severity (17). The absence of leukocytosis and the preservation of renal function further characterize this moderate biological profile, consistent with a subacute clinical presentation. These findings underscore the importance of integrating clinical, biological, and temporal data to distinguish NMS from isolated infectious conditions.

Such clinical courses have been reported in the literature as characteristic of situations in which a concomitant infection may mask or delay the identification of the initial expression of NMS (16). Indeed, Zhao et al. reported three cases of NMS complicated by pneumonia occurring shortly after the initiation or adjustment of a long-acting injectable antipsychotic, in a context of impaired consciousness, dysphagia, and hypersalivation suggestive of aspiration pneumonia (18). Similarly, a systematic review and meta-analysis including 683 cases of NMS identified respiratory complications as an independent predictor of mortality (19), highlighting the need for systematic respiratory screening and intensive monitoring in severe forms. These data confirm that pneumonia, far from being a mere diagnostic pitfall, represents a comorbidity of significant prognostic relevance in NMS.

Thus, this case illustrates the semiological and prognostic significance of pneumonia in neuroleptic malignant syndrome (NMS): beyond its role as a diagnostic confounder, it may represent a secondary systemic complication resulting from autonomic dysfunction, altered consciousness, and swallowing disorders. Early recognition of this pathogenic interaction requires an integrated multidisciplinary approach combining antibiotic therapy, correction of hydro-electrolyte disturbances, and close respiratory monitoring. Such a dynamic strategy optimizes both vital and functional outcomes in atypical forms of NMS associated with pulmonary infection.

The diagnosis of neuroleptic malignant syndrome (NMS) remains particularly challenging, especially in its atypical forms, due to symptomatic overlap with other neuropsychiatric conditions. Malignant catatonia represents a major differential diagnosis, particularly when it presents with mutism, negativism, and rigidity—clinical features closely resembling those observed in the present case (10).

However, the presence of fever, which is typically absent in pure catatonic states, favored the diagnosis of NMS in this case. Although catatonia induced by paliperidone palmitate (PP1M) is extremely rare, with only a single case reported in the literature (20), it nevertheless warrants consideration in the differential diagnostic process.

Serotonin syndrome also represents a significant diagnostic challenge due to its clinical similarity with neuroleptic malignant syndrome (NMS) (21), as both conditions may present with hyperthermia, autonomic instability, altered mental status, and neuromuscular abnormalities, exposing patients to potential misdiagnosis if pharmacological history and specific neuromuscular signs are not carefully assessed. In the present case, some initial clinical features—such as moderate fever (38 °C), tachycardia, profuse sweating, and confusion—could nonspecifically suggest excessive serotonergic stimulation.

However, several clinical and pharmacological factors formally exclude serotonin syndrome: the absence of exposure to serotonergic agents such as SSRIs or MAOIs (22), the predominance of generalized “lead-pipe” rigidity rather than myoclonus or hyperreflexia (21, 23), the close temporal correlation with the administration of paliperidone palmitate (24), and marked elevations in creatine phosphokinase and ferritin (23, 25), reflecting significant rhabdomyolysis typical of NMS. Furthermore, the prolonged course and gradual improvement following discontinuation of the dopaminergic antagonist (21) are more consistent with the pathophysiology of NMS than with serotonin syndrome, which typically resolves rapidly after cessation of the causative agent.

Additionally, other drug-induced extrapyramidal syndromes must be systematically excluded, including akathisia, parkinsonism, acute dystonias, and iatrogenic tremors (26). Recognition of NMS requires heightened vigilance regarding clinical and laboratory signs, particularly following the administration of long-acting antipsychotics, and a rigorous diagnostic approach in cases with atypical clinical presentations.

This clinical interpretation is closely aligned with the pharmacological understanding of paliperidone palmitate (PP1M), the active metabolite of risperidone, whose pharmacodynamic profile—characterized by predominant antagonism of dopamine D_2_ and serotonin 5-HT_2_A receptors, along with affinity for α_1_- and α_2_-adrenergic and H_1_-histaminergic receptors, and no documented anticholinergic or β-adrenergic activity (22, 23)—helps elucidate the pathophysiology of the observed syndrome. From a pharmacokinetic perspective, PP1M is distinguished by its prolonged release following intramuscular injection, with a half-life ranging from 25 to 49 days and a plasma peak observed around day 12 (24).

Within this framework, sustained D_2_ receptor antagonism induces a persistent reduction in central dopaminergic transmission, a key pathophysiological mechanism underlying neuroleptic malignant syndrome (NMS) (21). Central hypodopaminergia, particularly within the nigrostriatal, hypothalamic, and reticular circuits, accounts for the classical clinical constellation (rigidity, hyperthermia, altered consciousness, and dysautonomia) (21). Concurrently, tonic suppression of dopaminergic control over the sympathetic system may lead to adrenergic hyperactivity, responsible for autonomic instability (tachycardia, blood pressure fluctuations), a phenomenon amplified by the prolonged kinetics of PP1M (25). Thus, in patients treated with PP1M, the occurrence of NMS may be facilitated by this pharmacodynamic and pharmacokinetic profile, making early recognition of this risk particularly critical.

This case illustrates the kinetic particularities of NMS induced by paliperidone palmitate (PP1M). Unlike classical NMS, which typically arises acutely within days of initiating or increasing the dose of an oral neuroleptic, forms associated with long-acting injectable antipsychotics are characterized by delayed onset and prolonged course, directly related to the depot pharmacokinetics (27). In our observation, the use of monotherapy reinforces the causal attribution to PP1M, whose sustained release explains the atypical clinical course, far exceeding the timeframes classically reported with oral formulations.

Clinical improvement was observed only from day 10, while normalization of CPK levels was achieved only by day 35, reflecting a slow clinical and biological recovery fully consistent with NMS induced by long-acting formulations. This prolonged kinetic profile is comparable to that described with flupentixol decanoate, another long-acting injectable formulation (28). The continuous release of the active compound, persisting even after treatment discontinuation, limits the effectiveness of abrupt cessation and complicates symptomatic management, highlighting the specific challenges associated with long-acting antipsychotics.

The atypical clinical presentation, combined with a concomitant pneumonia, may have contributed to the initial diagnostic delay, thereby narrowing the optimal window for dopaminergic agonist administration. In the absence of a specific etiological treatment, management relied on a rigorous symptomatic approach, including parenteral rehydration, antipyretic therapy, targeted antibiotics, and intramuscular diazepam, which may have contributed to the attenuation of muscular hypertonia and improvement in the patient’s autonomic function.

This favorable, albeit gradual, clinical course demonstrates the relative efficacy of the intensive supportive strategy implemented in the management of atypical NMS in the absence of specific pharmacological treatment. Similarly, the gradual reintroduction of olanzapine was well tolerated and allowed the achievement of sustained clinical stability at six months, illustrating the feasibility of therapeutic switching following the resolution of PP1M-induced NMS (13).

Taken together, these findings align with the current conceptualization of neuroleptic malignant syndrome as a clinical continuum, in which atypical forms correspond to partial, subacute, or non-classical presentations. The current lack of consensual and clearly defined diagnostic criteria for atypical NMS constitutes a major limitation, exposing patients to potential underdiagnosis or delayed management in clinical practice. The convergence of clinical, biological, pharmacokinetic, and temporal data, consistent with recent literature, supports interpreting this observation within the neuroleptic malignant syndrome spectrum, characterized by a partial, subacute, and non-classical presentation, and underscores the importance of an integrative diagnostic approach for such subsyndromic manifestations.

## Conclusion

This case report highlights the diagnostic challenges posed by atypical NMS, particularly when induced by a long-acting antipsychotic such as paliperidone palmitate. The clinical course, characterized by partial symptomatology and prolonged biological abnormalities, reflects the impact of PP1M pharmacokinetics on the duration of neuroleptic malignant syndrome. The presence of a confounding factor, such as a concomitant infection, may obscure the iatrogenic etiology and delay appropriate management.

## Data Availability

The raw data supporting the conclusions of this article will be made available by the authors, without undue reservation.
